# Skeletal and dentoalveolar characteristics of maxillary lateral incisor agenesis patients: a comparative cross-sectional study

**DOI:** 10.1186/s12903-022-02656-7

**Published:** 2022-12-15

**Authors:** Mostafa A. Tageldin, Yomna M. Yacout, Eiman S. Marzouk

**Affiliations:** Department of Orthodontics, Faculty of Dentistry, Alexandria University, Champollion St., Azarita, P. O. Box: 21521, Alexandria, Egypt

**Keywords:** Maxillary lateral incisor agenesis, Congenitally missing teeth, Cephalometrics

## Abstract

**Background:**

The aim of the study was to evaluate the cephalometric and dentoalveolar characteristics of maxillary lateral incisor agenesis patients, and to compare the findings to a matched control group without tooth agenesis, excluding third molars, from the same population.

**Methods:**

The pre-orthodontic records of 72 non-growing patients, who were treated at the Orthodontic Department, Faculty of Dentistry, Alexandria University, were used to address the aim of this retrospective study. Patients having unilateral or bilateral maxillary lateral incisor agenesis, with no history of previous orthodontic treatment, congenital craniofacial malformations, facial trauma, or surgeries were divided into two test groups based on the pattern of maxillary lateral incisors agenesis (group I: unilateral (UMLIA), group II: bilateral (BMLIA)). A control group (group III (CTRL)) having a complete set of permanent dentition (excluding third molars), and having no dental anomalies was age-matched with the test groups. Measurements were performed on the pre-orthodontic lateral cephalometric radiographs and the pre-orthodontic digital dental casts. The measured variables were compared between the groups using one-way ANOVA and Kruskal Wallis tests according to the normality of the variable. In case of significant results, both tests were followed by multiple pairwise comparisons using Bonferroni adjusted significance level. Significance level was set at *P* < 0.05.

**Results:**

BMLIA group showed a smaller SNA angle and maxillary length, a more negative ANB angle and Wits appraisal, and a larger Maxillo-mandibular differential than UMLIA and/or CTRL group. The dental and soft tissue cephalometric measurements did not show any significant differences between the groups. Dentoalveolar cast measurements showed that BMLIA patients presented with significantly smaller maxillary inter-canine width than UMLIA and CTRL patients.

**Conclusions:**

Cephalometric analysis has shown that subjects with BMLIA have a statistically significant reduced ANB and maxillary length. Tooth eruption may play a role in the development of the maxillary arch.

## Background

Tooth agenesis refers to failure of formation of either deciduous or permanent teeth. The absence of teeth, whether congenital or otherwise, may result in arch length discrepancies, and occlusal disturbances [1, 2]. In addition, congenital hypodontia may be associated with craniofacial discrepancies, especially skeletal class III [3]. The facial profiles of hypodontia patients were found to be more concave than those of the general population [4].

The permanent maxillary lateral incisor is considered the most common congenitally missing tooth, excluding third molars [1, 5, 6]. The prevalence and pattern of congenitally missing permanent maxillary lateral incisors varies considerably among the different studies [1, 6–10]. Recently, Swarnalatha et al. [11] reported a prevalence rate of 3.77% in an orthodontic adolescent population aged 12–18 years, of which 62.16% had bilateral lateral incisor agenesis. The variation in the prevalence and in the pattern of agenesis may be related to the racial and ethnic differences between the different populations, the environmental effects, or the different sampling methods [1, 8, 11].

Early diagnosis and timely management of tooth agenesis is important to avoid potential problems such as alveolar bone atrophy and dental malocclusions [12]. Additionally, congenital absence of maxillary incisors may influence the skeletal pattern. Patients with congenitally missing maxillary lateral incisors were shown to have a marked skeletal class III tendency [13], primarily due to maxillary hypoplasia and retrusion [14]. Recently, Buyuk et al. [15] and Bassiouny et al. [9] reported decreased maxillary transverse and anteroposterior dimensions, respectively, in missing maxillary lateral incisor patients.

Early growth modification may be undertaken in maxillary lateral incisor agenesis cases as a preventive measure to enhance the growth of the maxilla [9]. Enhancing the growth of the maxilla may prevent future complications, such as development of anterior crossbite, and may facilitate future prosthetic replacement of the missing lateral incisors. However, the association between lateral incisor agenesis and the maxillary size and antero-posterior position is still not well-defined, and previous research [9] did not differentiate between unilateral and bilateral maxillary lateral incisor agenesis cases which warrants further investigation.

Hence, the purpose of the study was to evaluate the cephalometric and dento-alveolar characteristics of maxillary lateral incisor agenesis patients and to compare the findings to a matched control group without tooth agenesis, excluding third molars, from the same population. The null hypothesis of the current study was that there is no difference between maxillary lateral incisor agenesis patients and control patients regarding the cephalometric and dentoalveolar characteristics.

## Methods

A retrospective study design was used to address the aim of the study. Ethical approval (No. 0416-3/2022) was obtained from the institutional review board at the Faculty of Dentistry, Alexandria University (IRB: 00010556–IORG: 0008839). An exemption from requiring an informed consent was granted from the Research Ethics Committee of the Faculty of Dentistry, Alexandria University. The pre-orthodontic records of patients treated at the Orthodontic Department, Faculty of Dentistry, Alexandria University were screened for eligibility by the principal investigator to obtain the required sample size. Sample size was estimated using G*Power software (version 3.1.9.2., Universität Düsseldorf, Germany) assuming 80% study power and 5% α error. The minimum sample size was calculated to be 24 patients per group (total sample size 72 patients) based on a previous study [9] that reported mean maxillary length (Co-A) of 74.8 ± 7.2 mm in maxillary lateral incisor agenesis cases, and 80.6 ± 6.8 mm in control cases [16].

The inclusion criteria to select the eligible records were: (1) availability of complete pre-orthodontic records (case history, lateral cephalometric radiographs, panoramic radiographs, and dental casts), (2) unilateral or bilateral maxillary lateral incisor agenesis, (3) cessation of growth (judged from the lateral cephalometric radiographs using the cervical vertebrae maturation method) [17]. The exclusion criteria were: (1) previous orthodontic or orthopedic treatment, (2) patients with cleft lip and/or palate or congenital craniofacial malformations or diagnosed syndromes, (3) history of facial trauma or surgery, (4) history of extraction of permanent teeth, (5) poor quality of radiographs. To maintain the confidentiality of the participants, all the records were coded by the principal investigator (E.M.). Only the principal investigator had access to any potentially identifying patient information.

Diagnosis of congenitally missing maxillary lateral incisors was performed by one researcher (E.M) based on the pre-orthodontic panoramic radiographs. Congenital absence was asserted when no evidence of the tooth could be found on the radiograph, and the case history confirmed that no extractions had been performed. The same researcher and another researcher (Y.Y.) re-examined ten randomly selected records two weeks after the initial assessment, and 100% agreement in diagnosis of congenital absence was obtained between both researchers.

The selected records were divided into two test groups based on the pattern of maxillary lateral incisors agenesis: Unilateral maxillary lateral incisor agenesis group (UMLIA) and Bilateral maxillary lateral incisor agenesis group (BMLIA). A third group, having a complete set of permanent dentition (excluding third molars), no dental anomalies, minor or no dental crowding, and facial harmony, was matched with the test groups based on age, and acted as a control (CTRL).

**Fig. 1:**
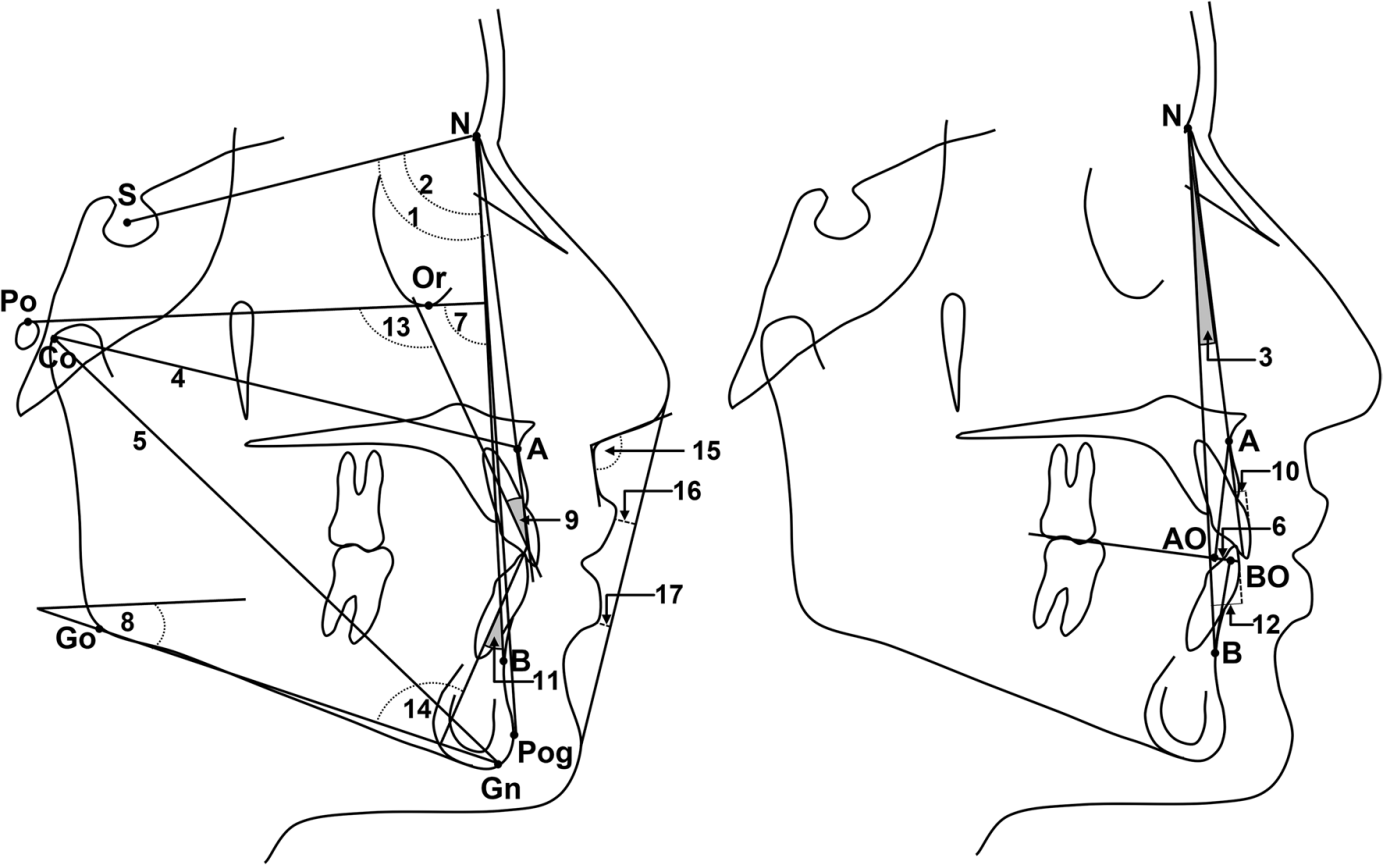
Skeletal, dental, and soft tissue cephalometric landmarks and measurements: (1) SNA; (2) SNB; (3) ANB; (4) Co-A; (5) Co-Gn; (6) wits appraisal; (7) facial angle; (8) mandibular plane angle; (9) U1/NA angle; (10) U1/NA distance; (11) L1/NB angle; (12) L1/NB distance; (13) U1/FH; (14) L1/Md plane; (15) Nasolabial angle; (16) U lip/E-line; (17) L lip/E-line. (See Table 2 for definitions of the measured parameters)

**Fig. 2:**
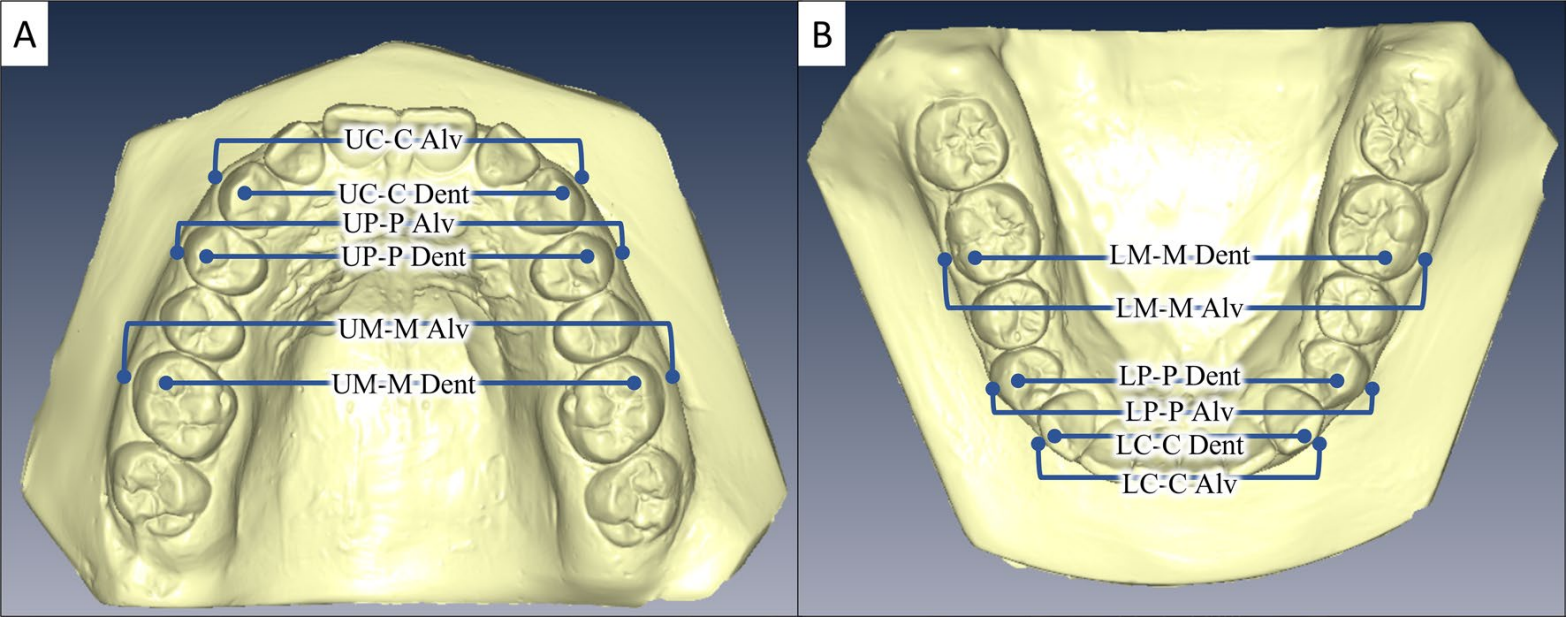
Maxillary and mandibular dento-alveolar cast measurements. (See Table 3 for abbreviations)

The lateral cephalometric radiographs were traced, and measurements were performed by one investigator (M.T.) using the cephalometric module in Blue sky plan® software (Blue Sky Bio LLC, Grayslake, Ill). The measured parameters are defined in Table 1 and depicted in Fig. 1 [9]. The researcher was blinded to the group assignment during assessment of the lateral cephalometric radiographs. The stone dental casts were digitized using inEos X5® scanner (Sirona Dental Systems GmbH, Bensheim, Germany) and exported in STL file format using inLab® software (Sirona Dental Systems GmbH, Bensheim, Germany). Transverse dento-alveolar measurements shown in Fig. 2 and Table 2 were performed on the digital dental casts by one investigator (Y.Y.) using Viewbox software, version 4.0.1.7 (Kifissia, Greece) [15]. Blinding was not possible when performing measurements on the dental casts because the presence or absence of the lateral incisor could be ascertained from the casts. All the cephalometric tracings and dental cast measurements were verified by another investigator (Y.Y. and M.T., respectively) and any disagreements were resolved by discussing and finding a mutually agreed upon landmark. The linear and angular parameters were measured to the nearest 0.01 mm and 0.1°, respectively.

**Table 1:** Skeletal, dental, and soft tissue cephalometric measurements

Measurement	Definition
*Skeletal measurements*	
SNA	The angle between the Sella-Nasion (S–N) plane and the Nasion-A point (N-A) line
SNB	The angle between the SN plane and the Nasion-B point (N-B) line
ANB	The angle between the N-A line and the N-B line
Co-A	Maxillary length measured from Condylion (Co) to A point
Co-Gn	Mandibular length measured from Co to Gnathion (Gn)
Maxillo-mandibular differential	The difference between maxillary length (Co-A) and mandibular length (Co-Gn)
Wits appraisal	The distance between two points that are formed by dropping perpendicular lines from A point and B point to the occlusal plane (AO and BO, respectively)
Facial angle	The inferior inside angle in which the facial line (N-Pog) intersects the Frankfort horizontal (Po-Or) plane
Mandibular plane angle	The angle between the mandibular plane (Tangent to Gonial angle and Menton) and Frankfort horizontal plane
*Dental measurements*	
U1/NA angle	The angle formed by the intersection of the N-A line with a line passing through the incisal edge and root apex of the maxillary central incisor
U1/NA distance	The distance between the maxillary central incisor and N-A line
L1/NB angle	The angle formed by the intersection of the N-B line with a line passing through the incisal edge and root apex of the mandibular central incisor
L1/NB distance	The distance between the mandibular central incisor and N-B line
U1/FH	The angle formed by the intersection of the Frankfort horizontal plane with a line passing through the incisal edge and root apex of the maxillary central incisor
L1/Md plane	The angle formed by the intersection of the mandibular plane with a line passing through the incisal edge and root apex of the mandibular central incisor
*Soft tissue measurements*	
Nasolabial angle	The angle formed by drawing a line tangent to the base of the nose and a line tangent to the upper lip
U lip/E-line	The distance between the upper lip and the esthetic line (extending from soft tissue tip of the nose to the soft tissue chin point)
L lip/E-line	The distance between the lower lip and the esthetic line (extending from soft tissue tip of the nose to the soft tissue chin point)

**Table 2:** Maxillary and mandibular dento-alveolar cast measurements

Maxillary inter-canine dental width (UC-C Dent)	The distance between the cusp tips of the right and left maxillary canines
Maxillary inter-canine alveolar width (UC-C Alv)	The distance between two points at the muco-gingival junctions above the cusp tips of the right and left maxillary canines
Maxillary inter-premolar dental width (UP-P Dent)	The distance between the buccal cusp tips of the right and left maxillary first premolars
Maxillary inter-premolar alveolar width (UP-P Alv)	The distance between two points at the muco-gingival junctions above the buccal cusp tips of the right and left maxillary first premolars
Maxillary inter-molar dental width (UM-M Dent)	The distance between the mesiobuccal cusp tips of the right and left maxillary first molars
Maxillary inter-molar alveolar width (UM-M Alv)	The distance between two points at the muco-gingival junctions above the mesiobuccal cusp tips of the right and left maxillary first molars
Mandibular inter-canine dental width (LC-C Dent)	The distance between the cusp tips of the right and left mandibular canines
Mandibular inter-canine alveolar width (LC-C Alv)	The distance between two points at the muco-gingival junctions above the cusp tips of the right and left mandibular canines
Mandibular inter-premolar dental width (LP-P Dent)	The distance between the buccal cusp tips of the right and left mandibular first premolars
Mandibular inter-premolar alveolar width (LP-P Alv)	The distance between two points at the muco-gingival junctions above the buccal cusp tips of the right and left mandibular first premolars
Mandibular inter-molar dental width (LM-M Dent)	The distance between the mesiobuccal cusp tips of the right and left mandibular first molars
Mandibular inter-molar alveolar width (LM-M Alv)	The distance between two points at the muco-gingival junctions above the mesiobuccal cusp tips of the right and left mandibular first molars

### Statistical analysis

To assess intra-examiner reliability, 5 records were randomly selected from each group using the RANDBETWEEN function in Microsoft® Office Excel software (Microsoft Corporation, Redmond, WA). All the measurements were repeated on the selected records after two weeks by the same researcher who performed the initial measurements. Intra-class correlation coefficients (ICC) were calculated.

Data were analyzed using Statistical Package for Social Sciences (SPSS) software for Windows version 23.0 (IBM Corp., Armonk, NY) and significance was inferred at *P* < 0.05. Normality was tested for all variables using plots (histogram and Q–Q plots), descriptive statistics, and normality tests. Means and standard deviation (SD) were calculated for all quantitative variables, while frequencies and percentages were calculated for qualitative variables (gender). Chi-square test was used for comparison of gender between the three study groups. Comparisons of study variables were done using one-way ANOVA and Kruskal Wallis tests according to the normality of the variable. In case of significant results, both tests were followed by multiple pairwise comparisons using Bonferroni adjusted significance level.

## Results

The chronological age and sex distribution among the three groups is shown in Table 3. No significant difference was found between the mean age of UMLIA patients (19.53 ± 3.15), BMLIA (20.21 ± 3.55) patients, and CTRL patients (20.05 ± 3.31) (*p* = 0.76). Similarly, no significant difference was found between the groups regarding the sex distribution (*p* = 0.78).

**Table 3:** Age and sex distribution among the study groups

	UMLIA (n = 24)	BMLIA (n = 24)	CTRL (n = 24)	*P* value
Mean age (SD), years	19.53 (3.15)	20.21 (3.55)	20.05 (3.31)	0.76^†^
*Sex*				
Male, n (%)	9 (37.5%)	7 (29.2%)	9 (37.5%)	0.78^‡^
Female, n (%)	15 (62.5%)	17 (70.8%)	15 (62.5%)	

BMLIA, bilateral maxillary lateral incisor agenesis; CTRL, control; SD, standard deviation; UMLIA, unilateral maxillary lateral incisor agenesis

^†^One-way ANOVA test

^‡^Chi-squared test

ICC for the measured variables ranged between 0.834 and 0.999 indicating good to excellent reliability [18].

Lateral cephalometric measurements of the three groups are shown in Table 4. Significant differences between the groups were found in six skeletal measurements (SNA, ANB, Co-A, Maxillo-mandibular differential, Wits appraisal, and Facial angle), while the dental and soft tissue measurements did not show any significant differences. The results of post-hoc pairwise comparisons of the lateral cephalometric measurements are presented in Table 5. Transverse dento-alveolar cast measurements showed that BMLIA patients presented with significantly smaller maxillary inter-canine dento-alveolar width than UMLIA and CTRL patients, and significantly smaller mandibular inter-canine dental width than the CTRL (Table 6). The results of post-hoc pairwise comparisons of the cast measurements are displayed in Table 7.

**Table 4:** Lateral cephalometric measurements in the three study groups

	UMLIA (n = 24)	BMLIA (n = 24)	CTRL (n = 24)	*P* value
	Mean (SD)			
*Skeletal measurements*				
SNA (°)	81.83 (4.66)^a^	79.32 (2.69)^b^	81.43 (3.41)^a,b^	0.04*
SNB (°)	77.88 (3.64)	79.34 (3.34)	78.80 (2.35)	0.28
ANB (°)	3.95 (2.72)^a^	− 0.01 (1.96)^b^	2.64 (1.98)^a^	< 0.001*
Co-A (mm)	83.81 (4.01)^a^	80.37 (3.10)^b^	85.48 (3.61)^a^	< 0.001*
Co-Gn (mm)	106.07 (6.62)	107.70 (5.68)	108.86 (4.68)	0.24
Maxillo-mandibular differential (mm)	22.26 (4.08)^a^	27.33 (5.09)^b^	23.38 (4.21)^a^	0.001*
Wits appraisal (mm)	0.70 (2.14)^a^	− 2.52 (3.54)^b^	− 0.70 (2.79)^a,b^	0.005*
Facial angle (°)	84.39 (3.71)^a^	88.51 (3.91)^b^	88.13 (3.43)^b^	< 0.001*
Mandibular plane angle (°)	26.30 (4.26)	24.01 (5.38)	25.03 (4.91)	0.27
*Dental measurements*				
U1/NA angle (°)	25.32 (6.16)	24.51 (6.03)	24.33 (8.28)	0.87
U1/NA distance (mm)	4.15 (2.25)	5.16 (2.59)	4.20 (2.50)	0.28
L1/NB angle (°)	29.53 (7.07)	26.84 (5.57)	27.20 (3.68)	0.20
L1/NB distance (mm)	5.75 (2.76)	5.21 (2.29)	5.41 (2.17)	0.74
U1/FH (°)	116.53 (6.64)	117.62 (6.02)	117.80 (4.71)	0.72
L1/Md plane (°)	93.48 (7.87)	92.68 (6.71)	93.40 (6.41)	0.91
*Soft tissue measurements*				
Nasolabial angle (°)	109.32 (9.71)	108.39 (10.48)	109.48 (9.59)	0.92
U lip/E-line (mm)	− 1.65 (3.17)	− 3.49 (2.53)	− 2.66 (2.91)	0.14
L lip/E-line (mm)	0.32 (2.89)	− 0.27 (2.73)	− 0.83 (1.91)	0.30

BMLIA, bilateral maxillary lateral incisor agenesis; CTRL, control; SD, standard deviation; UMLIA, unilateral maxillary lateral incisor agenesis

One-way ANOVA was used for all comparisons except ANB, Wits appraisal and U lip/E-line were compared using Kruskal Wallis test

*Statistically significant at *p* < 0.05

^a,b^Different letters denote statistically significant differences between groups using Bonferroni adjusted significance level

**Table 5:** Post-hoc pairwise comparisons of lateral cephalometric measurements between the three study groups

Variable	Group	Compared to	*P* value
SNA	Unilateral	Bilateral	0.01*
		Control	1.00
	Bilateral	Control	0.15
ANB	Unilateral	Bilateral	< 0.001*
		Control	0.59
	Bilateral	Control	0.001*
Co-A	Unilateral	Bilateral	0.004*
		Control	0.33
	Bilateral	Control	< 0.001*
Maxillo- mandibular differential	Unilateral	Bilateral	0.001*
		Control	1.00
	Bilateral	Control	0.01*
Wits	Unilateral	Bilateral	0.004*
		Control	0.23
	Bilateral	Control	0.43
Facial angle	Unilateral	Bilateral	0.001*
		Control	0.002*
	Bilateral	Control	1.00

*Statistically significant differences using Bonferroni adjusted significance level

**Table 6:** Cast measurements in the three study groups

	UMLIA (n = 24)	BMLIA (n = 24)	CTRL (n = 24)	*P* value
	Mean (SD)			
UC-C Dent	30.68 (2.57) ^a^	28.41 (2.78) ^b^	34.39 (2.30) ^c^	< 0.001*
UC-C Alv	35.55 (3.03) ^a^	32.67 (2.15) ^b^	37.80 (2.67) ^c^	< 0.001*
UP-P Dent	40.09 (2.62)	39.37 (3.17)	40.68 (1.96)	0.23
UP-P Alv	45.32 (2.19)	45.16 (3.27)	45.37 (3.05)	0.97
UM-M Dent	49.68 (3.00)	49.46 (3.09)	50.01 (3.02)	0.82
UM-M Alv	45.92 (2.97)	56.84 (2.92)	56.91 (3.30)	0.996
LC-C Dent	26.44 (2.27) ^a, b^	26.08 (1.66) ^a^	27.48 (2.04) ^b^	0.04*
LC-C Alv	31.58 (1.69)	30.92 (2.56)	32.03 (1.93)	0.19
LP-P Dent	35.11 (3.15)	34.27 (1.74)	35.27 (2.69)	0.37
LP-P Alv	40.89 (2.10)	40.60 (2.14)	40.49 (2.68)	0.83
LM-M Dent	44.02 (2.89)	44.15 (2.38)	44.49 (2.79)	0.83
LM-M Alv	54.04 (3.45)	54.36 (2.72)	53.97 (3.45)	0.90

BMLIA, bilateral maxillary lateral incisor agenesis; CTRL, control; SD, standard deviation; UMLIA, unilateral maxillary lateral incisor agenesis

One-way ANOVA was used for all comparisons

*Statistically significant at *p* < 0.05

^a,b,c^Different letters denote statistically significant differences between groups using Bonferroni adjusted significance level

**Table 7:** Post-hoc pairwise comparisons of cast measurements between the three study groups

Variable	Group	Compared to	*P* value
UC-C Dent	Unilateral	Bilateral	0.009*
		Control	< 0.001*
	Bilateral	Control	< 0.001*
UC-C Alv	Unilateral	Bilateral	0.001*
		Control	0.01*
	Bilateral	Control	< 0.001*
LC-C Dent	Unilateral	Bilateral	1.00
		Control	0.23
	Bilateral	Control	0.01*

*Statistically significant differences using Bonferroni adjusted significance level

## Discussion

Congenital absence of the maxillary lateral incisors is a common finding in orthodontic patients [1, 5, 6], with a higher prevalence of bilateral agenesis compared to unilateral agenesis [1, 5]. The skeletal pattern of the patients may be affected by maxillary incisors agenesis. Hence, in the current study, the lateral cephalometric radiographs and dental casts of lateral incisor agenesis patients were assessed to evaluate the relationship between incisor agenesis and the dimensions of the maxilla in the antero-posterior and transverse planes, respectively. An age-matched control group, which was selected from the same population, was used for comparison, and not the historic cephalometric norms because the dentofacial characteristics are affected by the patients’ ethnic backgrounds [19].

In the current study, congenital absence of maxillary lateral incisors was found to be more common in females than males when screening the orthodontic records for conformity with the eligibility criteria. This resulted in the sample being comprised of more females than males, however, statistical analysis did not show any significant difference between the groups. The increased occurrence of hypodontia in females more than males was previously reported in multiple studies [5, 6, 8, 9, 15, 20]. Nonetheless, the female predilection may be related to their increased motivation to seek orthodontic treatment for better smile esthetics [21].

In the current study, BMLIA patients presented with a significantly shorter maxillary length (Co-A), and SNA angle than UMLIA patients. Similarly, Co-A in the BMLIA group was significantly shorter than the CTRL group. Consequently, the difference between the maxillary and mandibular lengths was significantly larger in the BMLIA group compared to the other two groups, and the BMLIA patients showed a skeletal class III relationship (ANB = − 0.01 ± 1.96°, Wits appraisal = − 2.52 ± 3.54 mm) [22]. Analogous results were previously reported by Bassiouny et al. [9] in a sample of adult patients having congenitally missing lateral incisors. Based on Moss’s functional matrix theory, the underdevelopment of the maxillary bone may be related to the lack of a functional stimulation from the incisors [23].

No significant differences were found between the groups regarding the dental cephalometric measurements. The similarity of the dental inclination in the three groups suggests that there was no dental compensation in the BMLIA group despite the skeletal class III relationship. The expected dental compensation by proclination of the maxillary incisors is possibly offset by the absence of the lateral incisors which results in collapse of the maxillary arch anteriorly. This may also be related to the tendency of patients with hypodontia to have Angle class III dental malocclusions with anterior cross bites [5]. In contrast, Bassiouny et al. [9] found significant retroclination of the lower incisors in incisor agenesis patients which they attributed to dental compensation.

The nasolabial angle and the lips’ position relative to the E-Line did not show any statistically significant differences between the three groups despite the difference in the skeletal relationship. Contrarily, previous research reported a significantly larger nasolabial angle and more retrusive lips in missing lateral incisor patients compared to controls [9]. The results of the current study are supported by the findings of Fitzgerald et al. [24], where no correlation was found between the nasolabial angle and the skeletal measurements. Other factors, such as the angulation of the columella, play a role in the size of the nasolabial angle [24], hence the disparity between the studies.

The dental cast measurements showed that the maxillary inter-canine width and alveolar width were significantly smaller in the BMLIA group than in the UMLIA, which in turn was smaller than in the CTRL group. These findings are in agreement with previous research that investigated the transverse skeletal and dentoalveolar dimensions of the maxilla, in adolescent patients with lateral incisor agenesis, using posteroanterior cephalometric radiographs and dental casts [15]. The smaller dentoalveolar widths can be attributed to the eruption of the maxillary canines mesially into the place of the missing lateral incisors after shedding of their deciduous counterpart. Furthermore, presuming the deciduous incisors are not shed, their size is smaller than their permanent successors [25]. Additionally, unilateral maxillary lateral incisor agenesis is often accompanied with a microdontic contralateral incisor thus contributing to the decreased inter-canine dentoalveolar width [10]. In the current study, statistical analysis of the mandibular inter-canine width showed a significantly smaller width in the UMLIA and BMLIA groups compared to the CTRL group, however, the differences were not clinically significant. Previous research did not find any significant differences in the mandibular dentoalveolar measurements [15].

The early diagnosis of tooth agenesis is crucial to allow development of the alveolar bone [26]. Hence, early intervention to enhance the growth of the maxilla in cases with bilateral congenitally missing lateral incisors may avoid the development of skeletal class III malocclusion and may evade more invasive interventions later in life. Early treatment using reverse pull headgear may be part of the armamentarium of agenesis treatment.

### Limitations

One limitation of the current study is that the studied sample was selected from a single center. In addition, the retrospective cross-sectional nature of the study may be considered a methodological limitation because of its inability to infer a causal relationship. A longitudinal study design that evaluates the maxillary skeletal and dentoalveolar changes over time in patients with congenitally missing lateral incisors may better demonstrate the relation between lateral incisor agenesis and the maxillary dimensions. However, such a study design raises ethical concerns. Finally, it was not possible to blind the researcher during assessment of the transverse parameters because the presence or absence of the lateral incisors was evident from the dental casts. Nonetheless, blinding was performed during the analysis of the data.

## Conclusion

1. Cephalometric analysis has shown that subjects with bilateral maxillary lateral incisor agenesis have a statistically significant reduced ANB angle and maxillary length.
2. Tooth eruption may play a role in the development of the maxillary arch.

## Data Availability

The datasets analysed in the current research are available at synapse.org, under the title: Congenitally missing lateral incisors, https://doi.org/10.7303/syn45704342.

## Abbreviations

BMLIA: Bilateral maxillary lateral incisor agenesis
CI: Confidence intervals
CTRL: Control
SD: Standard deviation
UMLIA: Unilateral maxillary lateral incisor agenesis
